# Do feeding responses of a non-native bivalve outperform the native one in a coastal lagoon? A possible explanation for the invasion success of the dark false mussel *Mytilopsis leucophaeata*

**DOI:** 10.7717/peerj.15848

**Published:** 2023-08-18

**Authors:** Nathalia Rodrigues, Danielle Ribeiro, Igor C. Miyahira, Samira G. M. Portugal, Luciano N. Santos, Raquel A. F. Neves

**Affiliations:** 1 Graduate Program in Neotropical Biodiversity (PPGBIO), Institute of Biosciences (IBIO), Federal University of the State of Rio de Janeiro (UNIRIO), Rio de Janeiro, Brazil; 2 Research Group of Experimental and Applied Aquatic Ecology, Department of Ecology and Marine Resources, Institute of Biosciences (IBIO), Federal University of the State of Rio de Janeiro (UNIRIO), Rio de Janeiro, Brazil; 3 Laboratory of Chemical and Environmental Analysis, Institute of Biosciences (IBIO), Federal University of the State of Rio de Janeiro (UNIRIO), Rio de Janeiro, Brazil; 4 Laboratory of Theoretical and Applied Ichthyology, Department of Ecology and Marine Resources, Institute of Biosciences (IBIO), Federal University of the State of Rio de Janeiro (UNIRIO), Rio de Janeiro, Brazil

**Keywords:** Invasive species, Coastal lagoon, Clearance rate, Ingestion rate, Seston, Experimental ecology, Dreissenidae

## Abstract

The present study aimed to evaluate and compare feeding responses of the non-native and native bivalves, the dark false mussel *Mytilopsis leucophaeata* and the scorched mussel *Brachidontes darwinianus*, respectively, by offering different concentrations of seston from the coastal lagoon where these species coexist after dark false mussel introduction (Rodrigo de Freitas Lagoon, Rio de Janeiro-Brazil). For this purpose, independent laboratory experiments were carried out under five concentrations of seston to test the differences in clearance and ingestion rates of bivalves as a function of increasing concentrations of suspended particulate matter (SPM) on seston. In addition, from the integrated analysis of data obtained in experiments, it can be inferred about the efficiency levels of these species to remove SPM from seston and their effects on water turbidity and nutrient concentrations (total carbon, nitrogen, and phosphorus). Our hypothesis was that the non-native bivalve is more efficient to clear and ingest SPM from seston compared to the native one, which may lead to competitive advantages to the successful invasion of *M. leucophaeata* in coastal lagoons. Native species did not show a significant difference in clearance and ingestion rates with increasing concentrations of seston. Whereas the non-native bivalve showed a slight tendency to increase its clearance and ingestion rates with the increase in seston concentrations, evidencing its plasticity to adjust its feeding responses. The native bivalve was significantly more efficient to clear and ingest SPM at the lower seston concentration (*i.e*., close to natural concentrations found in the lagoon) compared to the non-native bivalve, which, on the other hand, showed a significant increase in its ingestion rates at the higher concentration tested (140 mg SPM L^−1^). Thus, the present results did not suggest food competition between the non-native *M. leucophaeata* and the native *B. darwinianus* in the introduced system. However, *M. leucophaeata* increased its feeding response with experimental increment in seston concentration, which suggests species ability to benefit from conditions of increased inputs of organic matter and higher primary production that could mediate its establishment in introduced systems.

## Introduction

Biological invasions are a widespread global problem, challenging the conservation of biodiversity and resources (Simberloff et al., 2013). Coastal marine ecosystems, including bays, estuaries, and lagoons, are often heavily impacted by human activities, including intentional and unintentional introduction of non-native species (Crooks, 2001). One of the main reasons is the release of non-indigenous species through ballast water of ships affecting coastal environments with ports infrastructure, especially at large cities with intense commercial activity by navigation (Minchin & Gollasch, 2002; Fernandes, Salgueiro & Miyahira, 2022). Impacts of non-native species in invaded systems often include the loss of native biodiversity induced by new-established ecological interactions in receiving community, the shifts in community structure and functioning, and possibly the changes in ecosystem physical and chemical properties (*e.g*., Anil, 2006; Florin et al., 2013; Kalchev et al., 2013; Ojaveer et al., 2018; Neves et al., 2020).

Dreissenid bivalves are among the most notable fresh- and brackish water invaders including, respectively, species of *Dreissena* (*D. polymorpha* and *D. bugensis*) and *Mytilopsis* (*M. leucophaeata* and *M. sallei*) (*e.g*., Vanderploeg et al., 2001; Verween, Vincx & Degraer, 2010; McLaughlan & Aldridge, 2013; Geda et al., 2018; Fernandes, Salgueiro & Miyahira, 2022). Most dreissenid studies are focused on the impacts of zebra mussels (*D. polymorpha*) and quagga mussels (*D. bugensis*) on freshwater systems, since these species are considered aggressive freshwater invaders that profoundly affect ecological and economic aspects of the introduced waterbodies (reviewed in Vanderploeg et al., 2001; Karatayev et al., 2021). However, despite the geographical spreading of non-native species of *Mytilopsis* in brackish systems worldwide (Rodrigues et al., 2022), little is known about its impacts on invaded coastal systems. Invasive *Mytilopsis* in brackish environments are known to tolerate wide ranges of temperature and salinity (*e.g*., Rajagopal et al., 2005; Verween, Vincx & Degraer, 2007, 2010; Astudillo, Bonebrake & Leung, 2017; Sa-Nguansil & Wangkulangkul, 2020). Moreover, free-living planktonic larvae and high fecundity of dreissenids facilitate species transfer to new habitats and rapid colonization (Bij de Vaate et al., 2002). In recent decades, new introductions of the dark false mussel *M. leucophaeata* has grown substantially in association with increased long-range ship displacements and economic globalization (Heiler, Nahavandi & Albrecht, 2010; Zhulidov et al., 2021; Rodrigues et al., 2022). The major vectors of dark false mussel transportation to non-native brackish systems are possibly hull fouling and inadequate management of ballast water (Fernandes et al., 2018). *Mytilopsis leucophaeata* is a native species in the Gulf of Mexico and the Atlantic Coast of United States of America, being a non-native species in coastal ecosystems in South America (Brazil, Venezuela, and possibly French Guiana), Eurasia (from Spain to Iran), and possibly in North Africa (Lodeiros et al., 2019; Minchin & Cottier-Cook, 2020; reviewed in Rodrigues et al., 2022).

In the last years, new records of the non-native dreissenids *M. leucophaeata* and *M. sallei* increased in Brazilian costal systems, with special attention to Northeast (Souza, Rocha & Lima, 2005; Farrapeira, Ferreira & Tenório, 2010; Galeão & Souza, 2015; Queiroz et al., 2020, 2022) and Southeastern coasts (Rizzo et al., 2014; Fernandes et al., 2020). In Rodrigo de Freitas Lagoon, an urban coastal system in Southeastern Brazil (Rio de Janeiro city), the dark false mussel *M. leucophaeata* was first recorded in 2014 (Rizzo et al., 2014). Currently this species is the most abundant epibenthic macrofauna in the lagoon, being widely distributed throughout its littoral zone (Maia-Neto, Caetano & Cardoso, 2020; Rodrigues et al., 2021). The evaluation of historical data of Rodrigo de Freitas Lagoon and results obtained from laboratory experiments evidenced that dark false mussels increased water transparency and reduced the density of fecal bacteria *Escherichia coli* (Neves et al., 2020). As previously described for other dreissenids, the feeding mechanism of these bivalves can induce direct increases in water clarity and light penetration in the water column (Cloern, 1982; Sousa, Gutiérrez & Aldridge, 2009; Cerco & Noel, 2010), that may affect phytoplankton physiology (Guildford et al., 2013), shift phytoplankton abundance and composition by selective grazing (*e.g*., size and prey type; Arnott & Vanni, 1996; Naddafi, Pettersson & Eklöv, 2007; Neves et al., 2020), and change nutrient dynamics (Arnott & Vanni, 1996; Naddafi, Pettersson & Eklöv, 2008). The gregarious behavior and high biomass production of *M. leucophaeata* also led to benthic macrofauna association to its aggregates in the lagoon, where nine taxa among Crustacea (*Cassidinidea fluminensis*, *Sinelobus stanfordi*, *Melita mangrovi*, *Eurypanopeus disimilis*, *Amphibalanus* spp.), Bivalvia (*B. darwinianus*), Gastropoda (*Heleobia* sp.), Polychaeta (*Alitta succinea*) and Diptera (Chironomidae larvae) was found using the hard substrate provided by dark false mussels as habitat (Rodrigues et al., 2021). However, the distribution of populations of the non-native and native bivalves in this coastal lagoon, respectively *M. leucophaeata* and *Brachidontes darwinianus*, suggested agonistic relationships (*e.g*., competition) between these species (Rodrigues et al., 2021).

Bivalves are filter-feeders that graze on suspended organic particles (*e.g*., microorganisms, phytoplankton, organic matter) and may alter ecosystem functioning due to their physiological responses playing an important role in nutrient cycling (Newell & Koch, 2004). The removal of suspended matters from the water column by bivalves converts organic particles into biomass and remobilizes organic matter to the benthic zone by the production of feces and pseudo-feces (Marescaux et al., 2016). Therefore, the present study aimed to evaluate whether the non-native dreissenid *M. leucophaeata* has a competitive advantage in obtaining food particles in Rodrigo de Freitas Lagoon by comparing its clearance and ingestion rates on suspended particulate matter (SPM) with feeding rates of the native bivalve *B. darwinianus*. For this purpose, controlled and independent laboratory experiments were carried out using different concentrations of seston from the coastal lagoon where these bivalve species co-occur to test differences on their feeding responses as a function of SPM in seston concentrations. Understanding the feeding behavior of bivalves can help to elucidate the role and effects of filter-feeding in the dynamics of aquatic ecosystems. Moreover, our results will provide insights about the efficiency levels of these bivalves for SPM removal and potential competitive advantages with implications for the understanding of the successful invasion of *M. leucophaeata* in the lagoon.

## Materials and Methods

### Study area

**Figure 1:**
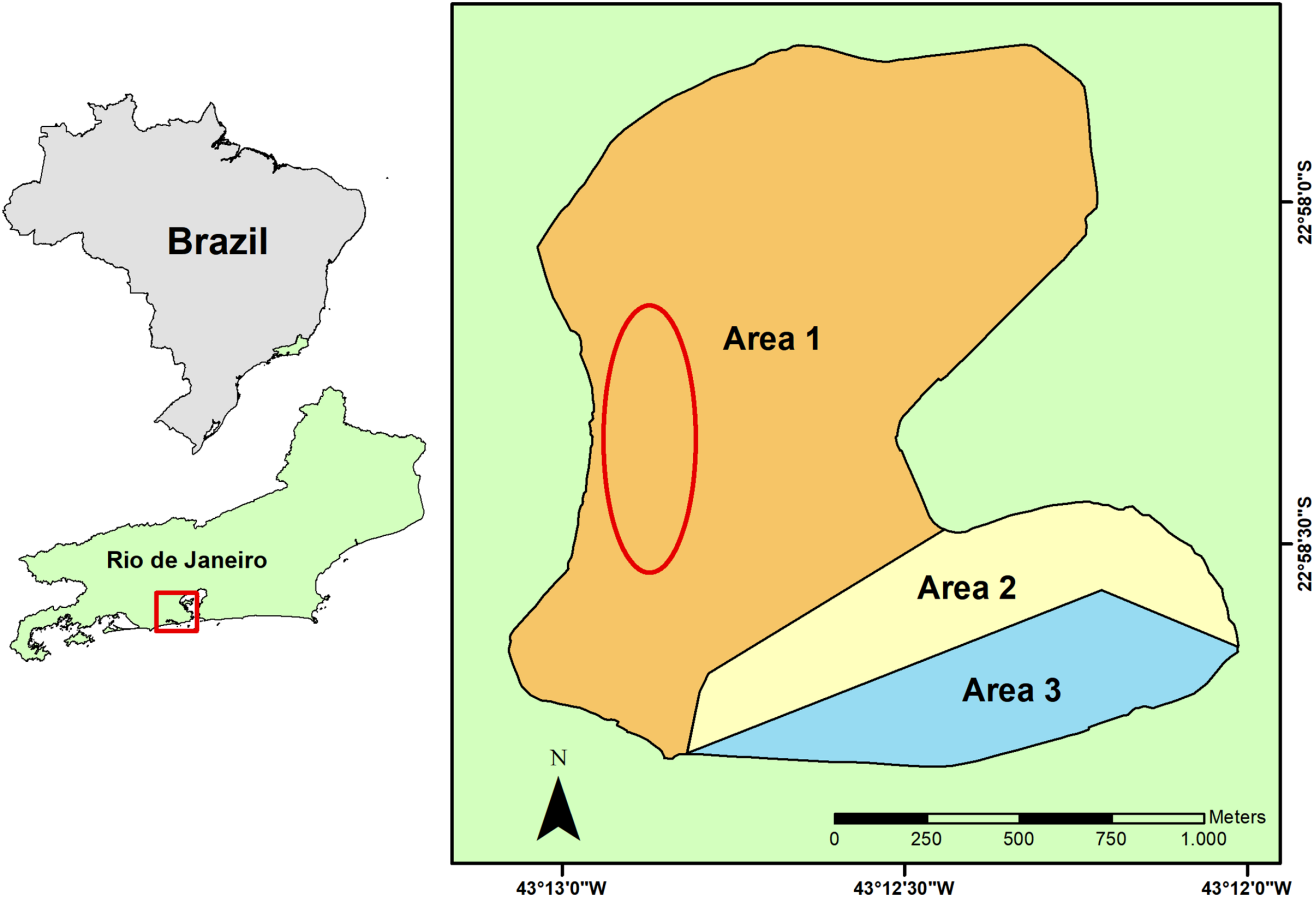
Geographical location of the Rodrigo de Freitas Lagoon, in the city of Rio de Janeiro, Southeastern Brazil. The sampling area of seston and non-native bivalves is indicated by a red ellipse, while the native bivalves were collected from hard substrata distributed in the whole littoral zone of the lagoon. The three different lagoon areas are indicated on the map by numbers and colors: 1 (white), 2 (yellow), and 3 (blue).

Rodrigo de Freitas Lagoon is a coastal shallow system of 2.2 km² located in an urban area at the South Zone of Rio de Janeiro city, Southeastern Brazil. This shallow costal lagoon is a semi-confined system with a complex dynamic of surface water renewal (Fonseca et al., 2014). Lagoon water conditions are mostly regulated by the inflow of rainwater and freshwater from its riverine system that includes the Macacos, Cabeça and Rainha rivers, and by the water exchange with the South Atlantic Ocean that sporadically enters the lagoon (Neves et al., 2020; Neves & Santos, 2022). Three floodgates artificially control the water level and the hydrological regime of the lagoon, both for freshwater and seawater, sustaining its brackish condition (Neves & Santos, 2022). Three areas (Fig. 1) were set in the Rodrigo de Freitas Lagoon by the municipal regulation n° 18145/2000, regarding its water circulation and environmental conditions, for the implementation of water monitoring programs and delimitation of regions for leisure and boat activities.

The phytoplankton community of Rodrigo de Freitas Lagoon is mainly composed of cyanobacteria, with dominance of picoplanktonic cyanobacteria (≤1.5 µm) (*e.g*., *Synechocystis aquatilis* and *S. salina)*, followed by diatoms (*e.g*., *Chaetoceros tenuissimus*, *Cyclotella* sp.) and cryptophytes (*e.g*., coccoid green alga) (Neves et al., 2020). The high abundance of cyanobacteria has been related to the influx of nutrients to the release of untreated sewage from houses, hospitals, and other establishments on its surroundings (Neves & Santos, 2022). In addition, the high densities of fecal bacteria frequently detected in the water—total coliforms and *Escherichia coli*—are evidence of illegal and continuous inputs of domestic sewage-derived pollutants into the lagoon (Neves et al., 2020).

### Sampling of seston

The sampling of seston (*i.e*., phytoplankton, microorganisms, detritus, organic and inorganic suspended material) from Rodrigo de Freitas Lagoon was carried out on July 12, 2021 (dry season). Concentrated seston was obtained by repeated horizontal trawls using a phytoplankton net of 20 µm mesh size. Horizontal trawls of surface water (~30 min in the total) were carried out on a motorized vessel covering an area (~1.5 km of extension) within the larger area of lagoon (area 1) to reduce food item variability (Fig. 1). Samples of concentrated seston were pre-filtered in stainless steel mesh (180 µm) to remove large particles outside bivalve’s ingestion range and other organisms (*i.e*., zooplankton) (Dalpadado, Ingvaldsen & Hassel, 2003) that could feed on organic particles in the samples. After that, concentrated seston from Rodrigo de Freitas Lagoon were maintained in sterilized transparent glass bottles (1 L) and kept on ice to preserve organic particles and organisms until its use in laboratory experiments.

A thermosalinometer (HI98319; Hanna Instruments, Smithfield, RI, USA) was used to determine the temperature (°C) and salinity (ppt) at the sampling to apply the average values found in lagoon under the laboratory experiments. Additionally, water samples (*n* = 3) were collected in sterilized amber glass flasks (250 mL) and kept on ice for the determination of total, organic and inorganic suspended particulate matter (mg L^−1^), and calculation of organic content (%) in the water of Rodrigo de Freitas Lagoon. Data of phytoplankton community was acquired from the weekly report of the municipal government that periodically monitor water quality at Rodrigo de Freitas Lagoon (Rio de Janeiro Municipality, 2021). During seston sampling, phytoplankton reached a density of 1.8 × 10^4^ cells mL^−1^ and its community was composed of cyanobacteria (43%), dinoflagellates (15%), dictiochophytes (12%), cryptophytes (10%), and other groups (20%) (Rio de Janeiro Municipality, 2021). During the sampling, there was no record of algal bloom and/or proliferation of harmful algal species that could affect feeding responses of bivalves.

### Sampling of native and non-native bivalves

Sampling of bivalves was conducted in accordance with the Chico Mendes Institute for Biodiversity Conservation (ICMBio n° 56897-6) and the National System for the Management of Genetic Heritage and Associated Traditional Knowledge (SISGEN n° A692B64). Individuals of *M. leucophaeata* (*n* = 100) and *B. darwinianus* (*n* = 38) were manually collected with a spatula from artificial (*e.g*., piers—the organisms were adhered to the pier, at low depth, close to the water surface) and natural substrates at Rodrigo de Freitas Lagoon, mostly concentrated in the area 1 of lagoon (Fig. 1), where both species co-occurred. However, a drastic reduction in the density of the native species was noticed during sampling, which required a greater sampling effort in terms of time (~10 h) and an expansion of sampling area throughout the whole perimeter of lagoon (areas 1, 2 and 3). Bivalves were placed into 20 L containers with lagoon water for transportation to the laboratory. Before the assays, bivalve shells were cleaned and measured (in length) using a digital calliper with 0.05 mm precision. Bivalves with total length varying from 15 to 20 mm ( $\overline {X\; }$ = 0.23 ± 0.06 g wet weight) were sorted to be used in the assays, since this size range did not differ in tissue mass and comprises the most representative cohorts of the bivalves found in Rodrigo de Freitas Lagoon (Maia-Neto, Caetano & Cardoso, 2020; Rodrigues et al., 2021). Subsequently, individuals of *M. leucophaeata* (*n* = 75) and *B. darwinianus* (*n* = 30) were acclimated under starvation for 48 h in glass aquariums (40 L) with artificial brackish water at controlled conditions of temperature (21.5 °C) and salinity (14 ppt), considering mean values of environmental conditions found at lagoon. Brackish water was produced using a commercial marine aquarium salt mix (Blue Treasure), that contains no nitrates and phosphates, and fresh distilled water until it reached the salinity measured at the Rodrigo de Freitas Lagoon during the sampling (14 ppt).

### Experimental design

**Figure 2:**
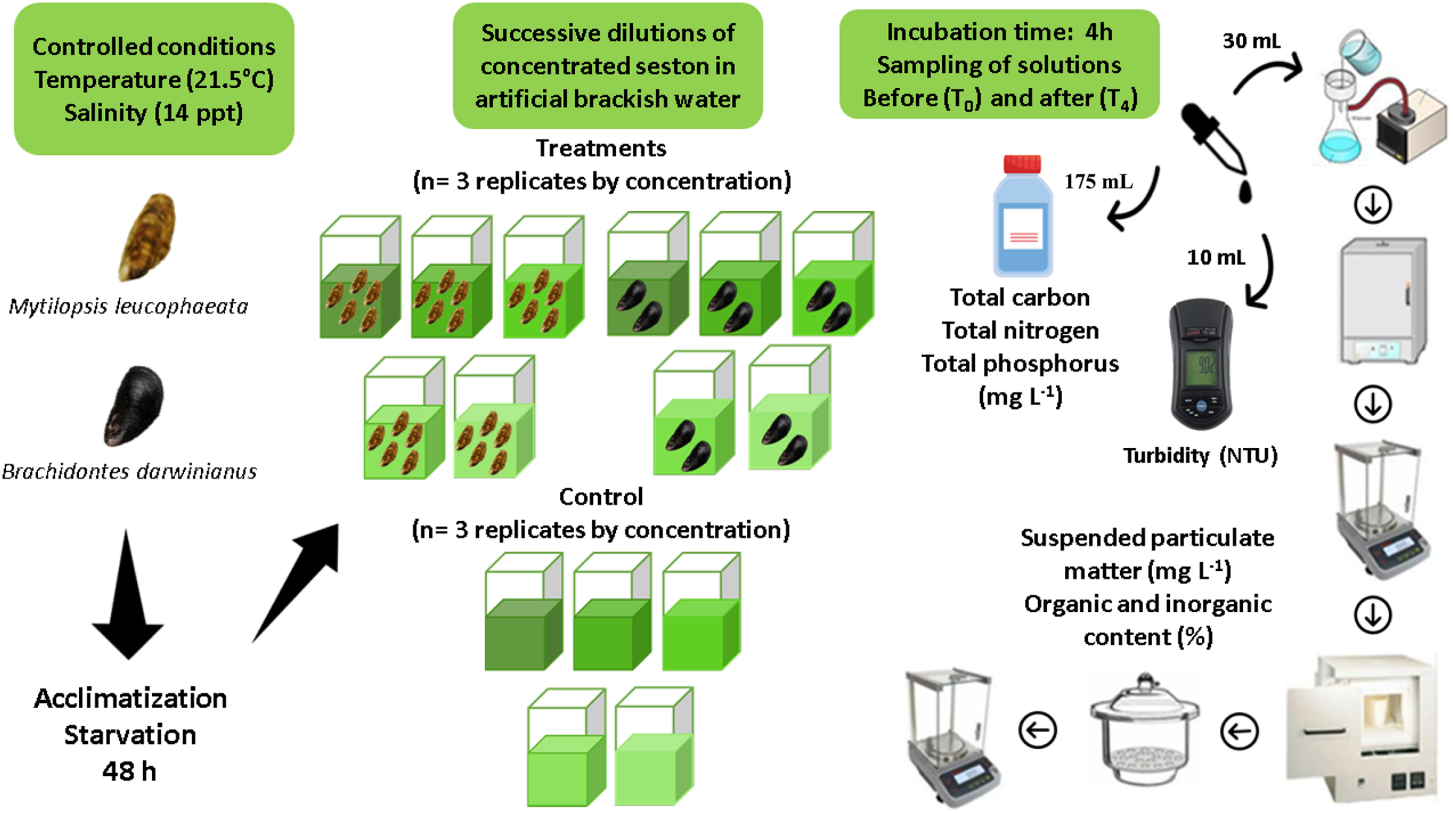
Experimental design indicating all the steps of sample’s processing for data acquisition. Short-term incubations (4 h) of the non-native and native bivalves *Mytilopsis leucophaeata* and *Brachidontes darwinianus*, respectively, and negative controls (without bivalves) in five treatments with different seston concentrations.

Assays consisted in short-term incubations (4 h) of five non-native bivalves (*M. leucophaeata*) and two native bivalves (*B. darwinianus*) per square glass aquariums (1 L) fulfilled with artificial brackish water and concentrations of seston (treatments) from Rodrigo de Freitas Lagoon. The maximum number of individuals per aquarium was chosen considering aquarium area (*i.e*., space) and seston concentration offered as food based on previous studies (Rodrigues et al., 2023), thus ensuring that no interference would occur among individuals within the replicates that could affect their feeding responses. Moreover, differences in the number of individuals by species treatment was corrected in feeding calculations; therefore, all study analyses could be performed using the same unit by variable. Aquariums (replicates) were kept under controlled conditions of salinity (14 ppt) and temperature (21.5 °C) with aeration to keep the concentrations of dissolved oxygen and food particles in suspension. Three independent replicates of treatment (*i.e*., incubation with bivalves) for native and non-native species and three independent replicates of negative control (*i.e*., incubation without bivalves) were performed for the five concentrations of seston during assays (107, 120, 127, 131, and 140 mg L^−1^). Negative controls were incubated under the same conditions as treatment’s replicates, to monitor fluctuations in the total suspended particulate matter (SPM) independently of bivalves. A detailed illustration of experimental design and laboratory procedures are shown as Fig. 2.

### Laboratory analyses

Aliquots of aquarium’s water (30 mL) were collected at the beginning and end of the incubation period, using a graduated glass serological pipette and automatic pipettor to determine the concentrations of total SPM, and the percentage of organic and inorganic content by replicates. Water samples were filtered through a carbon-free glass fiber membrane (MN, 0.7, 47 mm) using a vacuum pump attached to a filtration system. The glass fiber membranes were previously washed with distilled water, pre-combusted in a muffle at 450 °C for 3 h and weighed (W1) on an analytical balance (0.0001 g). After water filtration, the glass fiber membranes with retained particles (*i.e*., samples) were then carefully removed from the vacuum system and left for drying in an oven at 80 °C for 24 h. After that, samples were maintained in a desiccator with silica gel for cooling and subsequently weighted on analytical balance (*i.e*., dry weight—W2). Thereafter, samples were calcinated in a muffle at 450 °C for 1 h 30 min, kept in a desiccator with silica gel for cooling, and weighted again on analytical balance (*i.e*., weight after calcination—W3). Total SPM (mg L^−1^) and the percentages of organic and inorganic content (%) were calculated based on Navarro & Widdows (1997), as follows:

${\rm Total \;suspended\; particulate \;matter\; (SPM, mg L^{-1})}=\left( {\displaystyle{{\left( {W2 - W1} \right)} \over V}} \right)$

${\rm Organic\; content\; (\%) =}\left( {\left[ {\displaystyle{{\left( {W2 - W3} \right)} \over V}\; } \right] \times 100{\rm \; |}\; SPM} \right)$

${\rm Inorganic\; content \;(\%) =}\left( {\left[ {\displaystyle{{\left( {W3 - W1} \right)} \over V}\; } \right] \times \; 100\;{\rm |}\; SPM} \right)$

where, W1 = weight of pre-combusted glass fiber membrane (mg); W2 = dry weight of sample content retained in the glass fiber membrane (mg); W3 = weight of sample retained in the glass fiber membrane after calcination (mg); V = volume of filtered sample (L).

Additional aliquots of aquarium’s water (10 mL) were collected from each replicate of treatments and negative controls, at the beginning and end of incubations, for the determination of turbidity (NTU) using a turbidimeter (Instrutherm TD-300, ±0.5 NTU). Three analytical replicates were performed to assess turbidity of each water sample from experimental seston treatments, and the mean values were calculated.

Aliquots of water (175 mL) from each replicate were collected, at the beginning and end of incubations, in all the experimental replicates and controls for nutrient analyses. Samples were placed into glass flasks, previously washed with hydrochloric acid (HCl) solution (6 mol L^−1^), and preserved frozen in −20 °C until analyses. All the nutrient analyses followed standard protocols (APHA, 1998), total phosphorus (mg L^−1^) was determined using the ascorbic acid method, while total carbon (mg L^−1^) and total nitrogen (mg L^−1^) were analyzed using the combustion method in a total organic carbon analyzer (TOC-LCPH/CPN Shimadzu Corporation).

### Preparation of seston concentrations

Seston concentrations were obtained through an initial dilution of concentrated seston from the lagoon with artificial brackish water (one seston: three brackish water), followed by successive dilutions using a factor of 0.75. Five seston concentrations were achieved in treatments (107, 120, 127, 131, and 140 mg L^−1^), which represented a gradient of SPM ranging from 2.7 to 3.5 times higher than the suspended particulate matter found in the lagoon water during the sampling (*n* = 6; $\; \bar X$ ± SD = 42 ± 17 mg L^−1^). A slightly higher concentration of particulate matter in water suspension at the lower experimental concentration tested in comparison with the lagoon concentration was important to avoid total depletion of food particles in the first hours of incubation (Neves et al., 2020), allowing the calculation of feeding rates. A range of food depletion by bivalves from 15% to 30% during the incubation time are appropriate to calculate feeding rates in experimental studies (Mafra et al., 2009), therefore the present study considered pilot experiments and previous results obtained for the dark false mussels to adjust seston concentrations (Neves et al., 2020; Rodrigues et al., 2023).

**Figure 3:**
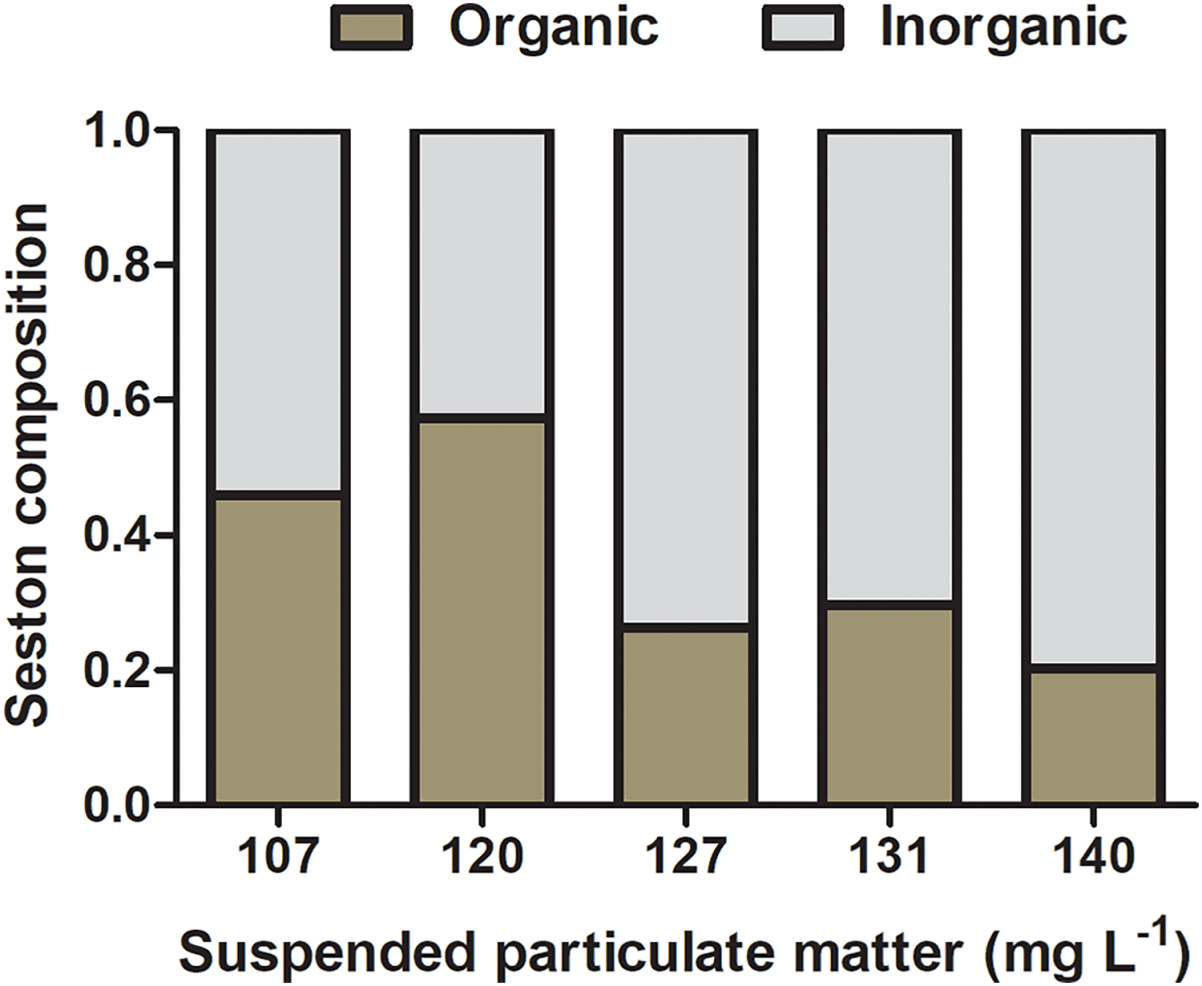
Initial composition of experimental seston. Organic (brown bar) and inorganic (gray bar) content by treatment (*i.e*., the concentration of suspended particulate matter on seston) before incubations with bivalves.

The composition of natural seston in the lagoon was 14 (±9.0) and 28 (±7.0) mg L^−1^ of organic and inorganic suspended matter, respectively, accounting for 33.3% of organic content and 66.6% of inorganic content. Concentrations of organic and inorganic particulate matter on seston at different experimental treatments varied from 39 and 68 mg L^−1^ and 51 and 103 mg L^−1^, respectively, and the percentage of organic content ranged from 20.3% to 57.4% among seston concentrations (Fig. 3). Considering the realistic approach applied to experiments using natural seston from the lagoon (*i.e*., live microorganisms, phytoplankton, detritus, organic and inorganic particles), a slight variation in the proportion of organic and inorganic content among different seston concentrations are expected and acceptable (as seen in Navarro & Widdows, 1997). However, considering that initial concentrations of seston were obtained in a volume that permitted the use in all the experimental replicates for a given concentration (*i.e*., incubations with both species and controls), there was no variation in the proportion of organic and inorganic content among replicates within the same seston concentration.

### Determination of clearance and ingestion rates

Clearance (mL ind^−1^ h^−1^) and ingestion (mg ind^−1^ h^−1^) rates of bivalves in response to seston availability were calculated using the difference in concentrations of SPM at the beginning and at the end of the incubation by replicate based on Coughlan (1969) and Riisgard, Kittner & Seerup (2003):

${\rm Clearance \;rate\; (mL \;ind^{-1} h^{-1}) =}\displaystyle{{K\times\; V} \over {n\; \times\; t}}$

${\rm Ingestion\; rate\; (mg\; ind^{-1} h^{-1}) =}{\rm Clearance\; rate\; }\times\left[ {{\rm SPM}} \right]$

where, K = (logarithmic variation between final and initial concentrations in experimental replicate) – (logarithmic variation between final and initial concentrations in treatment control); V = volume of suspension (mL); [SPM] = geometric mean of the initial and final concentrations of total suspended particulate matter in the replicate (mg L^−1^); n = number of individuals per replicate; and t = incubation time (h).

Data of turbidity and nutrients (total carbon, nitrogen, and phosphorus) were used, in percentage of control, as indicative of water quality variations induced by bivalves.

### Statistical analyses

Data normality and homogeneity of variances were assessed using Kolmogorov–Smirnov and Levene test, respectively, to verify the assumptions of parametric analysis. Proportion data of water turbidity and nutrients (in relation to controls) were transformed using arcsine of the square root to accomplish the assumptions for parametric test. A Generalized Linear Model (GML) was applied to test the effect of the interaction between seston treatment (*i.e*., seston concentrations) and bivalve species (native *vs*. non-native) on clearance rate, ingestion rate, and turbidity proportion. Additionally, univariate analyses—the parametric one-way ANOVA or the non-parametric Kruskal–Wallis—were applied to evaluate the effect of seston concentration (treatment) on ingestion rates, clearance rates, and water turbidity by bivalve species. When statistical analyses showed significant results (*p* ≤ 0.05), Tukey test or multiple comparisons were applied *a posteriori* of parametric and non-parametric tests, respectively. A t-test for dependent samples were applied to data of nutrients (*i.e*., variation in total carbon, total nitrogen, and total phosphorus) for comparisons between native and non-native bivalves by treatment (seston concentration). Statistical analyses were performed using the software Statistica 10.0 (StatSoft, Tulsa, OK, USA) and the graphs were developed using the software Graph Pad Prism 5.0.

## Results

**Figure 4:**
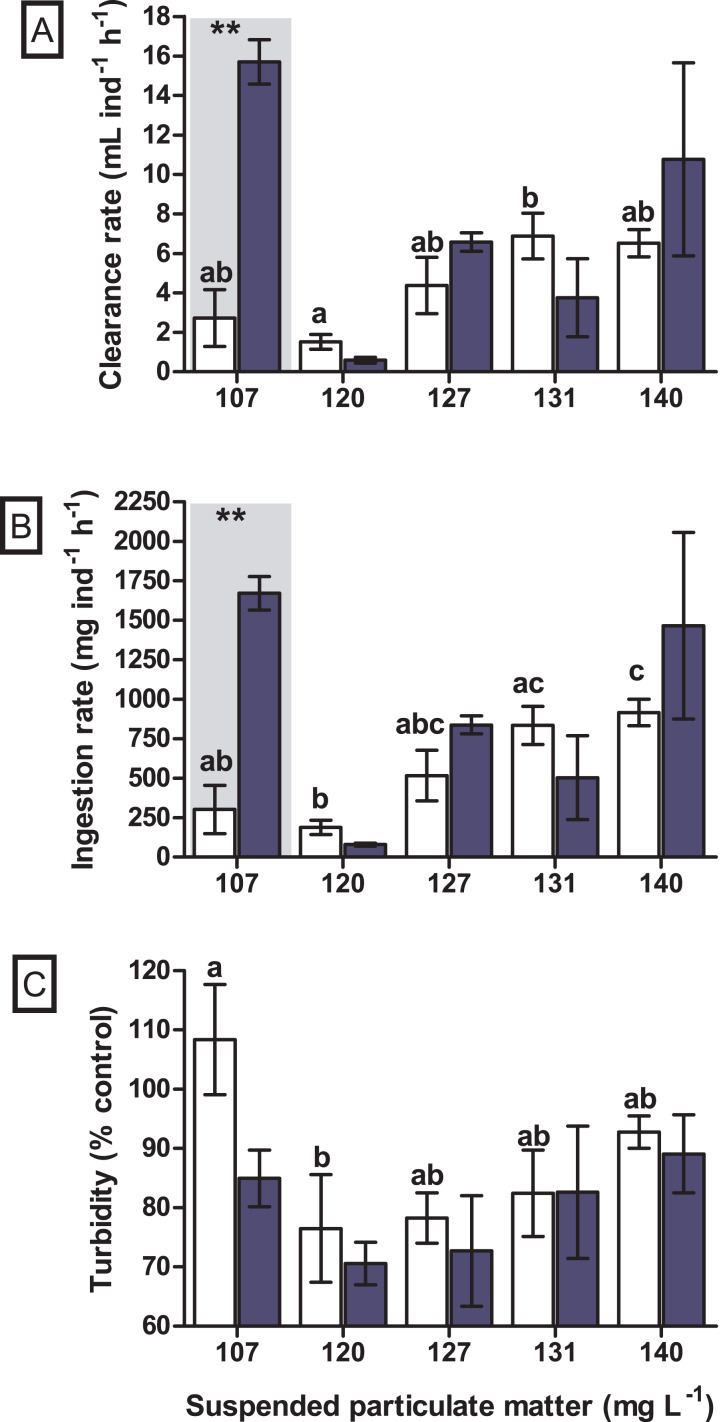
Feeding responses of the non-native and native bivalves, respectively *Mytilopsis leucophaeata* (white bars) and *Brachidontes darwinianus* (dark-blue bars), as a function of seston concentration. (A) Clearance rate (mL ind^−1^ h^−1^). (B) Ingestion rate (mg ind^−1^ h^−1^). (C) Turbidity variation (% control). Data are shown as means (± standard deviation) of three replicates by treatment, and seston concentration is expressed in suspended particulate matter (mg L^−1^). Bars in the hatched area indicate a statistically significant difference (**) between the bivalve species (*M. leucophaeata* and *B. darwinianus*) for a given concentration of seston (Tukey, *p* ≤ 0.05). Different letters indicate significant differences among seston concentrations for treatments with the non-native bivalve (*M. leucophaeata*).

A significant difference in the clearance rate was found for the interaction between seston concentration and species (GLM, F_4.29_ = 6.019, *p* = 0.002). When tested *a posteriori*, a significant difference in clearance rate between the bivalve species was observed at the concentration of 107 mg SPM L^−1^ (Tukey, df = 20, *p* = 0.004), in which the native bivalve *B. darwinianus* showed higher clearance rate when compared to the non-native *M. leucophaeata* (Fig. 4A). Univariate analysis evidenced a significant effect of seston concentration on clearance rate of the native bivalve *B. darwinianus* (Kruskal–Wallis, H_4.15_ = 9.29, *p* = 0.050). However, no significant difference was found using pairwise comparisons (multiple comparisons, *p* > 0.050), except for a marginally significant difference between the treatments of 107 and 120 mg SPM L^−1^ (multiple comparisons, *p* = 0.062) (Fig. 4A). While for the non-native bivalve *M. leucophaeata*, there was a significant effect of seston concentration on clearance rates (one-way ANOVA, F_4.10_ = 4.51, *p* = 0.024), in which the clearance rate observed at 120 mg SPM L^−1^ was significantly lower than the rate found in the treatment of 131 mg SPM L^−1^ (Tukey, df = 10, *p* = 0.039) (Fig. 4A).

Similarly, a significant difference in the ingestion rate was found for the interaction between seston concentration and bivalve species (GLM, F_4.29_ = 5.521, *p* = 0.004). When tested *a posteriori*, a significant difference in the ingestion rate between species was also observed at the concentration of 107 mg SPM L^−1^ (Tukey, df = 20, *p* = 0.007), in which the native bivalve showed higher ingestion rate compared to the non-native species (Fig. 4B). Univariate analysis applied to detect the effect of seston concentration on the ingestion rates of bivalve species evidenced similar results observed for clearance rates. A significant effect of seston concentration was detected on the ingestion rates of native bivalve (Kruskal–Wallis, H_4.15_ = 10.0, *p* = 0.040), but only a marginally significant difference in the ingestion rate of scorched mussels was found between the concentrations of 107 and 120 mg SPM L^−1^ (multiple comparisons, *p* = 0.062) (Fig. 4B). Ingestion rates of the non-native bivalve *M. leucophaeata* were significantly affected by seston concentration (one-way ANOVA, F_4.10_ = 7.0, *p* = 0.006), with significantly lower ingestion rate at 120 mg SPM L^−1^ compared to rates found in treatments of 131 mg SPM L^−1^ (Tukey, df = 10, *p* = 0.023) and 140 mg SPM L^−1^ (Tukey, df = 10, *p* = 0.031) (Fig. 4B).

**Figure 5:**
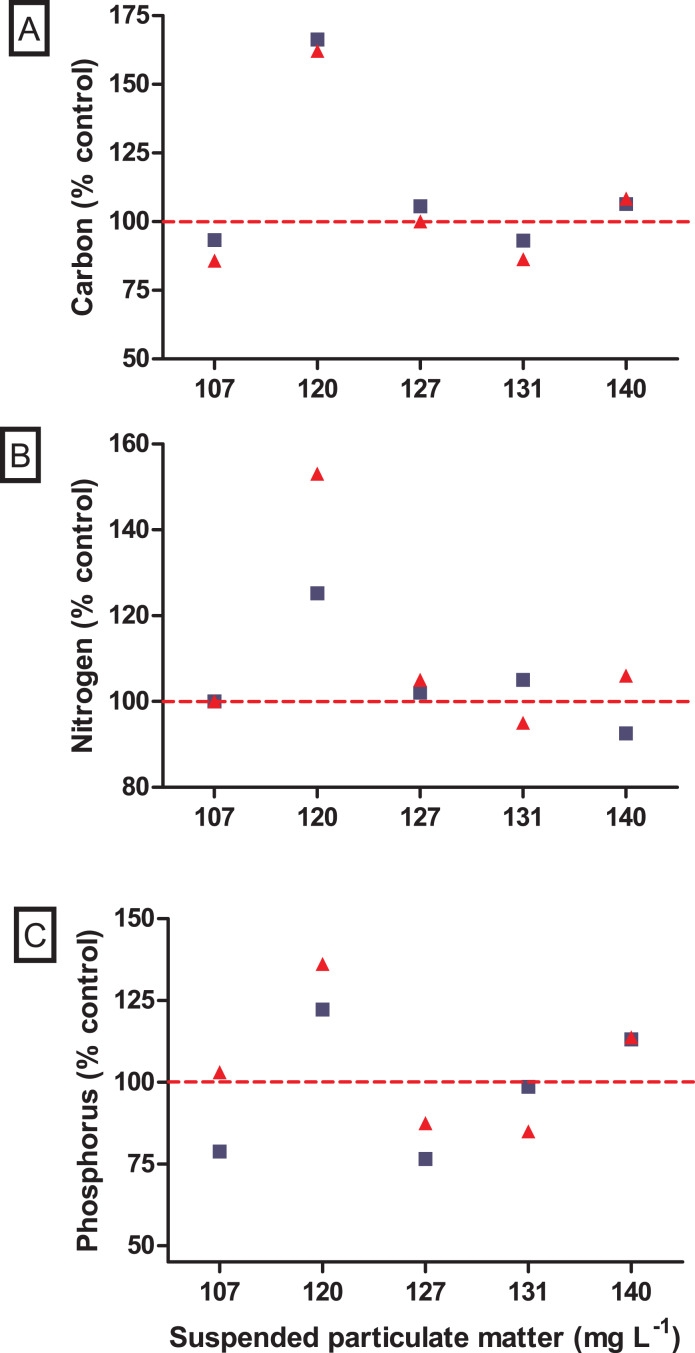
Nutrient variations after incubations with the non-native *Mytilopsis leucophaeata* (red triangle) and the native *Brachidontes darwinianus* (dark-blue square) by seston concentration, expressed in total suspended particulate matter (mg L^−1^). (A) Total carbon (% control). (B) Total nitrogen (%). (C) Total phosphorus (%). Data are provided as the mean values of nutrient variation in three replicates by seston concentration for each species. The dashed red line indicates 100% of compatibility between the concentrations obtained in treatments (with bivalves) and negative controls (without bivalves).

No significant difference was observed in the variation of water turbidity for the interaction between seston concentration and bivalve species (GLM, F_5.24_ = 0.946, *p* = 0.469). Despite the tendency of reduction in percentage of water turbidity (*i.e*., values lower than 100%) in treatments with the native bivalve *B. darwinianus*, no significant difference was detected among treatments (one-way ANOVA, F_5.17_ = 1.816, *p* = 0.184) (Fig. 4C). Variation in water turbidity was significantly affected in treatments with the bivalve *M. leucophaeata* (one-way ANOVA, F_5.17_ = 3.573, *p* = 0.033), particularly shown by the difference found between treatments of 107 and 120 mg SPM L^−1^. In the first scenario, an increase in water turbidity (*i.e*., greater than 100%) was detected and contrasted with the greater variation found at concentration of 120 mg SPM L^−1^ (*i.e*., lower turbidity compared to negative control) (Tukey, df = 12, *p* = 0.048) (Fig. 4C). Except for the incubation with *M. leucophaeata* at 107 mg SPM L^−1^, all the treatments showed a percentage of water turbidity lower than 100% (*i.e*., value in which turbidity in treatment would be equivalent to negative control), indicating that both native and non-native bivalves reduced water turbidity on seston compared to controls for a given SPM concentration. In terms of nutrients, total carbon, nitrogen, and phosphorus showed the higher concentrations for both species at the treatment of 120 mg L^−1^. No significant difference was found in the variation of total nitrogen (t-test, df = 4, *p* = 0.347) and total phosphorus (t-test, df = 4, *p* = 0.327) among treatments with native and non-native bivalves, except for a marginally significant difference detected for the variation in total carbon (t-test, df = 4, *p* = 0.06) (Fig. 5).

## Discussion

Laboratory experiments evidenced differences in feeding responses of native and non-native bivalves, respectively *B. darwinianus* and *M. leucophaeata*, that co-occur in Rodrigo de Freitas Lagoon. Scorched mussels were significantly more efficient at the lowest seston concentration tested (*i.e*., 107 mg SPM L^−1^) for clearance and ingestion rates compared with the non-native dark false mussel. Similarly, Galimany et al. (2018) evaluated the feeding responses of one native (*Ischadium recurvum*) and two non-native mussels (*Mytella charruana* and *Perna viridis*) in Florida (USA) and showed that invasive mussels have a similar feeding performance as the native mussel; however, considering the differences in habitat preferences of the three species, the invasive species do not fully overlap the native’s niche in the east coast of Florida. Another study carried out in Uruguay with the non-native freshwater bivalve *Corbicula fluminea* and the native *Diplodon parallelopipedon* showed lower feeding rates of non-native species when phytoplankton was composed of cyanobacteria, but the native bivalve kept similar rates, regardless of phytoplankton composition (Marroni et al., 2014).

In addition to organic particles of different nature, detritus, and inorganic particles, natural seston is also composed by phytoplanktonic organisms that are the basis of food for suspension-feeders. In the present study, phytoplankton of Rodrigo de Freitas Lagoon showed no dominance of any specific group (*i.e*., occurrence >50%) during the sampling of seston used in experiments and its community was composed of cyanobacteria, dinoflagellates, dictiophytes, and cryptophytes. Thus, in the present study, differences in feeding responses observed for the native and non-native bivalves do not seem to be related to phytoplankton composition on seston nor to seston quality, since both seston and bivalves used in incubations were collected at the same coastal system. It is important to highlight that, in our experimental study, it was tested a slightly higher concentration of particulate matter in water suspension in comparison with the lagoon concentration to avoid total food depletion allowing the calculation of feeding rates. Nevertheless, seston sampling and the analysis of suspended particulate matter in Rodrigo de Freitas Lagoon were conducted during dry season at Rio de Janeiro state (winter months: June–September), when a lower effect of inflows of rivers and pluvial waters is expected into the lagoon. Therefore, a higher content of particulate matter in this shallow coastal lagoon may be found during the rainy season (late spring and summer months: November–March) and/or after heavy rainfall events (Neves & Santos, 2022).

When analyzing the food concentration-dependent response by species, the non-native bivalve *M. leucophaeata* showed a slight tendency towards an increase in ingestion rates with increase in seston availability, showing a significant difference between the lower and higher seston concentrations. Increases in feeding response as a function of high food availability was observed for bivalves incubated with seston (Velasco & Navarro, 2002; Navarro & Velasco, 2003) and the microalga *Isochrysis galbana* (Rajesh, Mohamed & Krip, 2001). However, when analyzing the food concentration-dependent response of the native bivalve, *B. darwinianus* did not show significant differences in clearance and ingestion rates as a function of increase in seston concentration, and no significant variation in water turbidity was detected among treatments with native bivalve. This tendency of stabilization in the feeding of *B. darwinianus*, irrespectively of seston availability, suggests that the native species achieved its maximum rates at concentrations tested. In contrast, our experimental results indicate that the dark false mussel *M. leucophaeata* can adjust its feeding rates, increasing its ingestion rate in conditions with higher food availability, which could be an advantage for this non-native species in eutrophic lagoons. Moreover, *M. leucophaeata* can significantly remove suspended particulate matter from seston in experimental suspensions simulating hypereutrophic conditions (*i.e*., trophic state index >67), reaching its higher feeding rates close to 178 mg SPM L^−1^ (Rodrigues et al., 2023). A significant reduction in *M. leucophaeata* feeding rates was only noticed at the concentrations of 226 and 236 mg SPM L^−1^, which was suggested to be a stress response to higher concentrations of suspended particles (Rodrigues et al., 2023).

The ecological impacts of non-native species introduction in aquatic systems can be severe and of large proportions (Simberloff et al., 2013), but in the first moment their impacts are generally unpredictable and rarely detectable (Joyce, Kregting & Dick, 2019). A comparative study with bivalves evaluating algal uptake as resource use showed that the non-native *Crassostrea gigas* was less efficient in capture algal cells than the native *Mytilus edulis* (Joyce, Kregting & Dick, 2019). Although native species can be more efficient in the use of food resource, invaders that form dense populations can cause dramatic ecological impacts on community level, especially in aquatic environments with low water renovation (Newell, Wildish & MacDonald, 2001). Agglomerates of suspension feeding bivalves with high biomass can effectively regulate phytoplankton concentration and, consequently, chlorophyll *a* by filter-feeding (Dame et al., 2000), which can result in direct increases in water clarity and light penetration in the water column (Cloern, 1982; Sousa, Gutiérrez & Aldridge, 2009; Cerco & Noel, 2010; Neves et al., 2020). Moreover, dreissenid mussels may also contribute to high phosphorus inputs through excretion into introduced systems (Arnott & Vanni, 1996; James, Barko & Eakin, 2001; Conroy et al., 2005; Naddafi, Pettersson & Eklöv, 2008). In the present study, variations in water turbidity (*i.e*., lower values compared to negative controls, with a single exception) were coherent with the changes expected due to removal of suspended particles by the filter-feeder bivalves. In terms of nutrients, variations of total carbon, nitrogen and phosphorus did not differ between incubations with native and non-native bivalves. Nutrient variations in incubations with bivalves showed the same pattern for both species with values lower than or close to control; except for the concentration of 120 mg SPM L^−1^, in which carbon, nitrogen and phosphorus in incubations with bivalves were higher than in negative controls. In our experimental approach, natural microcosms with seston composed of live microorganisms (*e.g*., bacteria, picoplankton) might affect nutrient dynamics, particularly in incubations with bivalves that may change the nutrient bioavailability in incubations (including N:P ratios) through excretion (Vaughn & Hoellein, 2018; Neves et al., 2020). Therefore, possible changes in nutrient availability may favor opportunistic small phytoplankton (*e.g*., the frequent picocyanobacteria), as described in Vanderploeg et al. (2017), and heterotrophic microorganisms (*e.g*., bacteria, flagellates, and ciliates) (Neves et al., 2020). However, there is no evident explanation for this response been observed only at the concentration of 120 mg SPM L^−1^. Moreover, higher water transparency was significantly detected following *M. leucophaeata* introduction in Rodrigo de Freitas Lagoon (Neves et al., 2020). In this context, changes in water conditions carried out by the dense agglomerates of the non-native *M. leucophaeata* in Rodrigo de Freitas Lagoon may indirectly affect the fitness of the native species *B. darwinianus* by habitat modification (*e.g*., water quality).

Limitations regarding experimental studies are important to consider when results are scaled-up and used to broad implications. Despite the probable oversimplification of the environmental complexity found in shallow coastal lagoons, controlled experimental conditions allows, however, to test responses to one factor (*e.g*., food availability) and cause-and-effect relationships. Therefore, the present results highlight that the native bivalve *B. darwinianus* does not seem to be limited by food availability in the natural system, since its feeding responses are independent on food concentration, and scorched mussels are more efficient to clear and ingest suspended particulate matter from seston at concentrations close to the lagoon conditions (*i.e*., lower seston concentration tested). However, the invasive dark false mussel is the most abundant species of epibenthic macrofauna in Rodrigo de Freitas Lagoon, reaching densities between 60,000 to 120,000 ind. m^−2^ (Maia-Neto, Caetano & Cardoso, 2020), that are much higher than *B. darwinianus* in the same lagoon (Rodrigues et al., 2021). Environmental modifications induced by the dense aggregates and biomass dominance of *M. leucophaeata* may favor this non-native species over the native bivalve *B. darwinianus*. The wide tolerance of *M. leucophaeata* to abiotic conditions is expected to contribute to the successful establishment of this species in introduced systems (Rodrigues et al., 2022), but biotic relationships also seem to mediate its invasiveness potential. Several theories attempt to explain the success of non-native species, including the release of predators and pathogens, and the absence of competitors (*e.g*., Fagan et al., 2002; Simberloff et al., 2013). Rodrigo de Freitas lagoon provides optimal conditions of temperature and salinity for the establishment of the tropical lineage of *M. leucopheata* (Fernandes, Salgueiro & Miyahira, 2022), in addition the lagoon has few native competitors (*e.g*., scorched mussels, barnacles; Rodrigues et al., 2021) and potential predators (Moraes, Andreata & Oliveira, 2014), which seems to explain the massive colonization and distribution of dark false mussels in this coastal shallow lagoon. However, since no competitive advantage was detected in feeding behavior, other ecological interactions and biological responses (*e.g*., space competition, growth rates, reproductive rates, larval settlement) must be tested to confirm the successful mechanisms of *M. leucophaeata* in this introduced system to mitigate its negative impacts on native biota, especially the scorched mussel *B. darwinianus*.

## Conclusions

Feeding responses of the dreissenid bivalve *M. leucophaeata* assessed by clearance and ingestion rates of suspended particulate matter from seston did not suggest food competition between the non-native and native bivalve *B. darwinianus* in experimental conditions. Furthermore, the present results indicated a greater efficiency of the native bivalve to clear and ingest suspended particles at lower seston concentration compared to the non-native bivalve. Therefore, other ecological interactions and biological features of *M. leucophaeata* (*e.g*., reproductive rates, aggregation behavior or environmental resistance) make this invasive species the dominant filter feeder in this coastal lagoon. Further studies are needed to test other hypothesis of biological processes that may explain the suggested agonistic relationship between the native and non-native bivalves, as well as the high dominance and wide distribution of the dark false mussel *M. leucophaeata* in Rodrigo de Freitas Lagoon.

## Supplemental Information

10.7717/peerj.15848/supp-1 Supplemental Information 1 Raw bivalve feeding rate data. Clearance (mL ind^−1^ h^−1^) and ingestion (mg ind^−1^ h^−1^) rates of the bivalves *Brachidontes darwinianus* (B) and *Mytilopsis leucophaeata* (M) by suspended particulate matter (SPM) concentration (mg L^−1^).

10.7717/peerj.15848/supp-2 Supplemental Information 2 Raw nutrient data. Concentration (mg L^−1^) of total carbon, nitrogen, and phosphorus in treatments—control (C), native bivalve *Brachidontes darwinianus*, and non-native bivalve *Mytilopsis leucophaeata* (M).

10.7717/peerj.15848/supp-3 Supplemental Information 3 Raw water turbidity data. Turbidity (NTU) in treatments with the native and non-native bivalves, respectively *Brachidontes darwinianus* (B) and *Mytilopsis leucophaeata* (M), and negative control.

## Additional Information and Declarations

### Field Study Permissions

The following information was supplied relating to field study approvals (*i.e*., approving body and any reference numbers): Chico Mendes Institute for Biodiversity Conservation (ICMBIO) and National System for the Management of Genetic Heritage and Associated Traditional Knowledge (SISGEN) approved the study (ICMBio n° 56897-6; SISGEN n° A692B64).

### Data Availability

The following information was supplied regarding data availability: Raw data is available in the Supplemental Files.
